# Characterization and Performance Enhancement of Bio-Based Polyurethane-Modified Cement Mortar Utilizing Polyglycerol Polyester Polyol

**DOI:** 10.1021/acsomega.4c04228

**Published:** 2024-11-05

**Authors:** Renzo Miguel R. Hisona, Christine Joy M. Omisol, Tomas Ralph B. Tomon, Andrei E. Etom, Mike Jhun P. Calderon, Carlo Kurt F. Osorio, Dan Michael A. Asequia, Daisy Jane D. Erjeno, Ann Pearl G. Triana, Blessy Joy M. Aguinid, Adam Roy V. Galolo, Gerard G. Dumancas, Roberto M. Malaluan, Arnold C. Alguno, Arnold A. Lubguban

**Affiliations:** † Center for Sustainable Polymers, Mindanao State University – Iligan Institute of Technology, Iligan City 9200, Philippines; ‡ Department of Physics, Mindanao State University – Iligan Institute of Technology, Iligan City 9200, Philippines; § Manufacturing Engineering Technology Department, University of Science and Technology of Southern Philippines – Jasaan Campus, Katipunan Street, Lower Jasaan, Jasaan, Misamis Oriental 9003, Philippines; ∥ Department of Biology, College of Mathematics and Natural Sciences, Caraga State University, Ampayon, Butuan City 8600, Philippines; ⊥ Department of Chemistry, Loyola Science Center, The University of Scranton, Scranton, Pennsylvania 18510, United States; # Department of Chemical Engineering and Technology, Mindanao State University – Iligan Institute of Technology, Iligan City 9200, Philippines

## Abstract

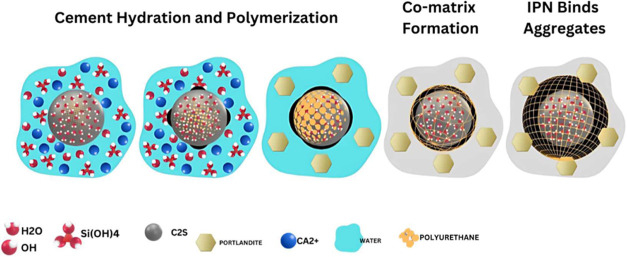

The increasing focus on sustainable construction is driving the industry toward materials that combine functionality with environmental benefits. A viable approach to address this demand is the use of bio-based additives to improve traditional cementitious composites. This study introduces a novel approach to developing a polymer-modified construction material by incorporating varied amounts (0, 1, 2, 3, and 6%) of bio-based polyurethane (PU), derived from polyglycerol polyester polyol, into cementitious mortar. The resulting PU-modified cementitious mortar (PUMC) was evaluated for its mechanical, physicochemical, and microstructural properties. Results show that the incorporation of 2% PU by cement weight significantly enhanced compressive strength by 58.2%, flexural strength by 37.0%, and initial flow performance by 20.0% after 28 days, while a 6% PU incorporation provided the best abrasion resistance. These improvements were attributed to a uniform particle and pore size distribution and the formation of a uniform interpenetrating polymer network (IPN), as confirmed by BET–BJH and SEM–EDX analyses. Additionally, FTIR and TGA analyses revealed that the metal–ligand coordination between Ca^2+^ ions in the cement mortar and PU ligand groups strengthened the interfacial connectivity through noncovalent bonding, further enhancing the material properties. This research highlights the potential of bio-based PU as an eco-friendly additive that significantly improves the performance of cementitious mortars, making it a promising option for industrial flooring applications.

## 1 Introduction

Industrial floorings are materials designed to withstand the unique demands of the industrial setting, including heavy machinery and equipment, foot traffic, and temperature variation.^1^ The addition of fillers,^2^ reinforcement,^3^ and polymer modification are among the commonly applied techniques to enhance the durability of industrial floorings. Out of these, polymer modifications such as polymer concrete (PC), polymer-modified concrete (PMC), and polymer-modified mortar (PMM) are among the most popular alternatives, as they offer a multitude of benefits in the industrial setting. It includes increased mechanical strength,^4,5^ durability,^6^ chemical resistance,^7^ and resistance to shrinkage and cracking,^8^ rendering it highly advantageous compared to ordinary mortar and concrete.^9^ The selection and addition of admixtures into the concrete design play a crucial role in altering or improving the overall performance and properties of the resulting polymer-modified cementitious materials.^4,5^ These admixtures have been frequently used in the past: epoxy,^10−13^ acrylic latex (AL), styrene–butadiene rubber (SBR),^5^ polyvinyl acetate (PVA), poly(vinylidene chloride) (PVC), and polyurethane (PU) and copolymers such as vinyl acetate/ethylene (VAE) and styrene-butyl acrylate (SBA).^14−16^ They are renowned for improving workability, increasing elastic modulus, and mechanical strength.^17−20^ Among these, the integration of polyurethane (PU) in structural and infrastructural engineering is deemed highly advantageous as it effectively enhances the composite’s Young’s modulus and improves its resistance against abrasion, bending, and ultimate elongation.^21,22^ Specifically, cement-based composites with PU exhibit superior strength and durability, toughness,^21^ and adhesion.

Although polymer modification is highly regarded for its contribution to the advancement of industrial flooring, several disadvantages have been presented. Notably, the integration of acrylic latex (AL) and styrene–butadiene rubber (SBR) not only significantly increased production costs but was also observed to decrease compressive strength.^22^ Additionally, the incorporation of poly(vinyl chloride) (PVC) into the concrete design has, in some cases, been reported to induce corrosion in steel.^23^ Another significant concern arises as these polymeric admixtures are petroleum-based. Statistics reveal that polymeric admixtures such as SBR, AL, PVC, PU, and other types of copolymers account for approximately 3% of global carbon emissions.^24,25^ For this reason, a paradigm shift favoring the utilization of bio-based polymer admixtures is essential for achieving more sustainable solutions in mortar and concrete modification. One of the most promising alternatives for this application is the production of PU from bio-based sources. Previous studies^26−28^ have demonstrated the successful synthesis of bio-based polyols to produce bio-based PUs. Furthermore, the effective integration of bio-based PUs into the concrete design^29−31^ highlights their potential as alternative sustainable polymer admixtures.

This research effort demonstrates the development of a bio-based polyurethane-modified cementitious mortar (PUMC) that utilizes sustainable polymer admixtures. The resulting PUMC was designed to improve the overall compressive and flexural strength, flow, and abrasion resistance. The physicochemical and mechanical properties of PUMC were assessed according to the American Society for Testing and Materials (ASTM) methods. The presence of the different functional groups in the composite material was analyzed by using a Fourier transform infrared spectrometer (FTIR) with attenuated total reflectance (ATR). The thermal behavior of the resulting composite was determined using thermogravimetric analysis (TGA) with derivative thermogravimetry (DTG). The specific surface area and pore structure were analyzed by using Brunauer–Emmett–Teller (BET) with Barrett–Joyner–Halenda (BJH). The surface morphology and elemental distribution were analyzed using scanning electron microscopy (SEM) with energy-dispersive X-ray spectroscopy (EDX). The physicochemical and mechanical test results indicated that the incorporation of bio-based PU significantly improved the self-leveling and mechanical properties of the modified cementitious mortar.

## 2 Materials and Methods

### 2.1 Materials

The proposed cement mortar mix design was prepared using Type I Portland cement produced by Holcim Philippines Incorporated (Lugait, Philippines). The superplasticizer and fine aggregates used were purchased locally. The fine aggregates utilized were refined salicaceous sand with an average bulk density of 2.35 g/cm^3^, sieved using a No. 200 sieve to attain a uniform size distribution. Ultrapure water was used in the mixture.^32^ The PPP used in this study was adapted from a previous study.^33^ The polymeric methylene diphenyl diisocyanate (MDI, PAPI 135 SH) was supplied by Chemrez Technologies, Inc. (Quezon City, Philippines). All of the materials mentioned were used as is and without any alteration. The characteristics of these components are presented in the following sections.

#### 2.1.1 Cement

Cement that satisfies the requirements of ASTM C150^34^ for Type I Portland cement was used as the fine aggregate binder. The chemical compositions of the cement used in this study are listed in Table 1.

**Table 1:** Chemical Composition of the Cement

SiO_2_	Al_2_O_3_	CaO	Fe_2_O_3_	MgO	SO_3_	loss on ignition
22.0	5.3	65.2	3.1	1.5	2.0	0.80

#### 2.1.2 Aggregate

**Figure 1:**
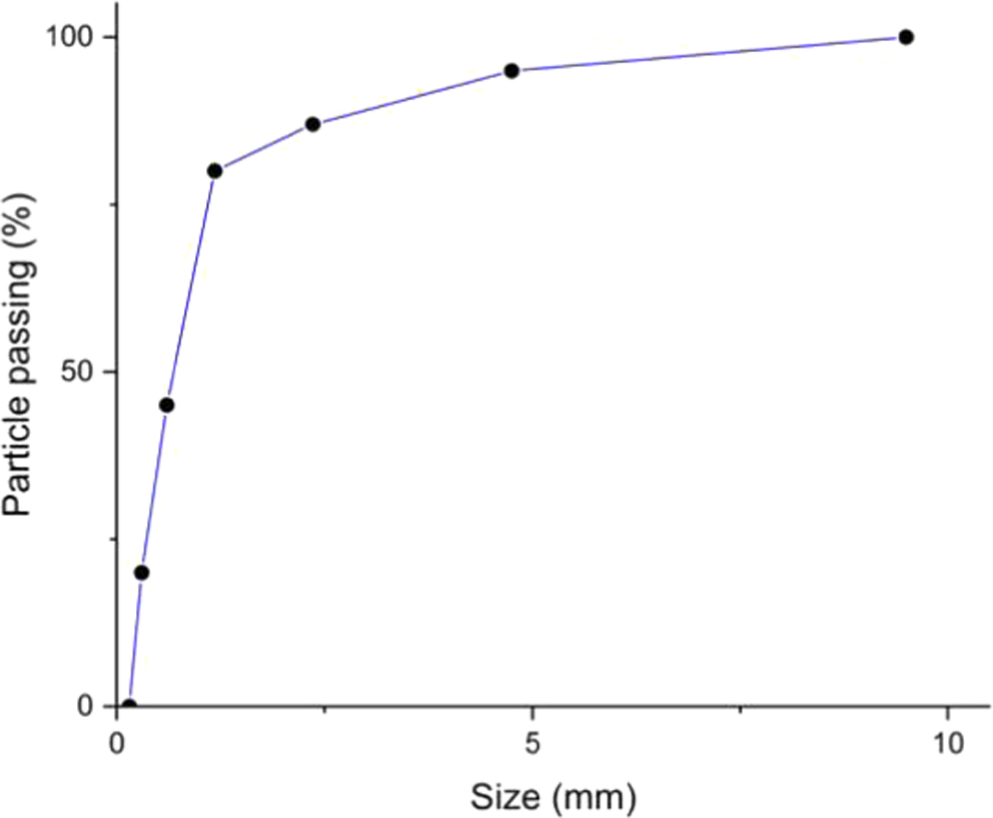
Sieve grading curve for the sand used as the fine aggregate.

Salicaceous sand satisfying the requirements for ASTM C128^35^ for Type A fine aggregate^36,37^ was used for preparing cement mortars with and without the PU admixture. The sieve grading^38^ for the sand used as fine aggregate is presented in Figure 1. The properties of the sand are listed in Table 2.

**Table 2:** Specific Gravity and Absorption of Fine Aggregates

fine aggregate	bulk saturated surface dry (SSD) specific gravity (g/cm^3^)	water absorption (%)
salicaceous sand	2.35	1.9

#### 2.1.3 Polyglycerol Polyester Polyol (PPP)

**Figure 2:**
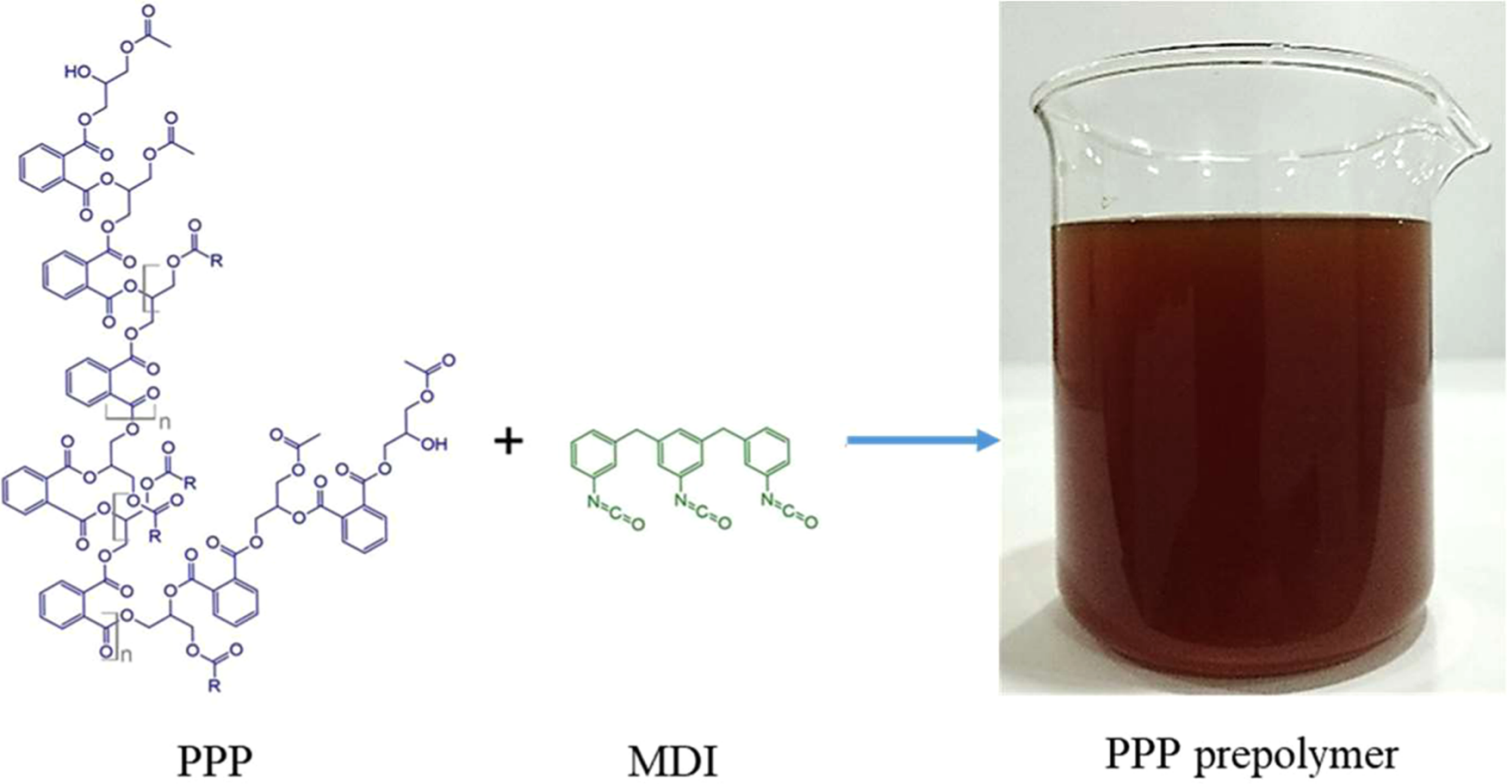
Polyglycerol polyester polyol (PPP) prepolymer used to produce bio-based polyurethane admixture for cement mortar.

The PU admixture used to produce the PUMC was obtained through the reaction of PPP and MDI (Figure 2). The details of the synthesis of PPP can be found in the previous study.^33^ The properties of the PPP used are listed in Table 3.

**Table 3:** Physical and Chemical Properties of Polyglycerol Polyester Polyol (PPP)

properties	PPP
appearance	dark brown viscous liquid
OH no. (mgKOH/g)	198
acid no. (mgKOH/g)	2–8
viscosity (mPa·s)	6180
molecular weight (g/mol)	4110

#### 2.1.4 Superplasticizer Admixtures

Self-leveling is an integral property for industrial flooring materials, hence, to impart this property to the modified cement mortar, 1 wt % of the cement^39,40^ of an SNFC-based superplasticizer^41^ was introduced to the PUMC. The physical and chemical properties of the superplasticizer are presented in Table 4.

**Table 4:** Physical and Chemical Properties of the Superplasticizer (SP)

properties	SP
chemical content	SNFC-based
form	powder
color	yellow brown
bulk density	0.45–0.65 g/cm^3^
solubility in water	insoluble
pH value	8.0–10.00

### 2.2 Methods

#### 2.2.1 Cement Mortar Mix Design, Sample Preparation, and Curing Conditions

The cement mortar mix design was prepared by varying the amount of PU in the mix according to the 1:0.5:0.35 proportion of cement, sand, and water listed in Table 5 to investigate its influence on the compressive and flexural strength of the cement mortar. For the preparation of the PUMC samples, a modified version of the component mixing system introduced by Ohama^42^ was adopted. In this system, a predetermined amount of cement and aggregate was weighed and dry blended in a mixing bowl at 300 rpm using a high-speed mixer.^43^ Then, the superplasticizer at 1 wt % by weight of cement was incorporated into the premix. Once homogenized, water^23^ was added and stirred continuously at 500 rpm for 4 min. Then, the PPP prepolymer was introduced into the mixture and was stirred for another 60 s. Finally, when fully integrated, a calculated amount of MDI was added and stirred for another 30 s. The resulting cement mortar mixtures were then poured into steel molds (50 × 50 × 50 mm^3^ for compressive strength and 40 × 40 × 160 mm^3^ for flexural strength) and covered with polyethylene sheets to prevent evaporation in the cement mortar mix.^44^ After 24 h, the cement mortar samples were removed from the steel molds and allowed to undergo dry curing at standard laboratory conditions, defined at 23.0 ± 2.0 °C, and relative humidity not less than 50%. Dry curing is a commonly adopted method for polymer-modified cement mortars, as this method is believed to be necessary for polymer film formation.^45^

**Table 5:** Cement Mortar Mix Design

	mass of components (g)				
specimen	cement	sand	water	SP	PU
0%PU/cement	100.00	50.00	35.00	1.00	
1%PU/cement	100.00	50.00	35.00	1.00	1.00
2%PU/cement	100.00	50.00	35.00	1.00	2.00
3%PU/cement	100.00	50.00	35.00	1.00	3.00
6%PU/cement	100.00	50.00	35.00	1.00	6.00

#### 2.2.2 Mechanical Properties

**Figure 3:**
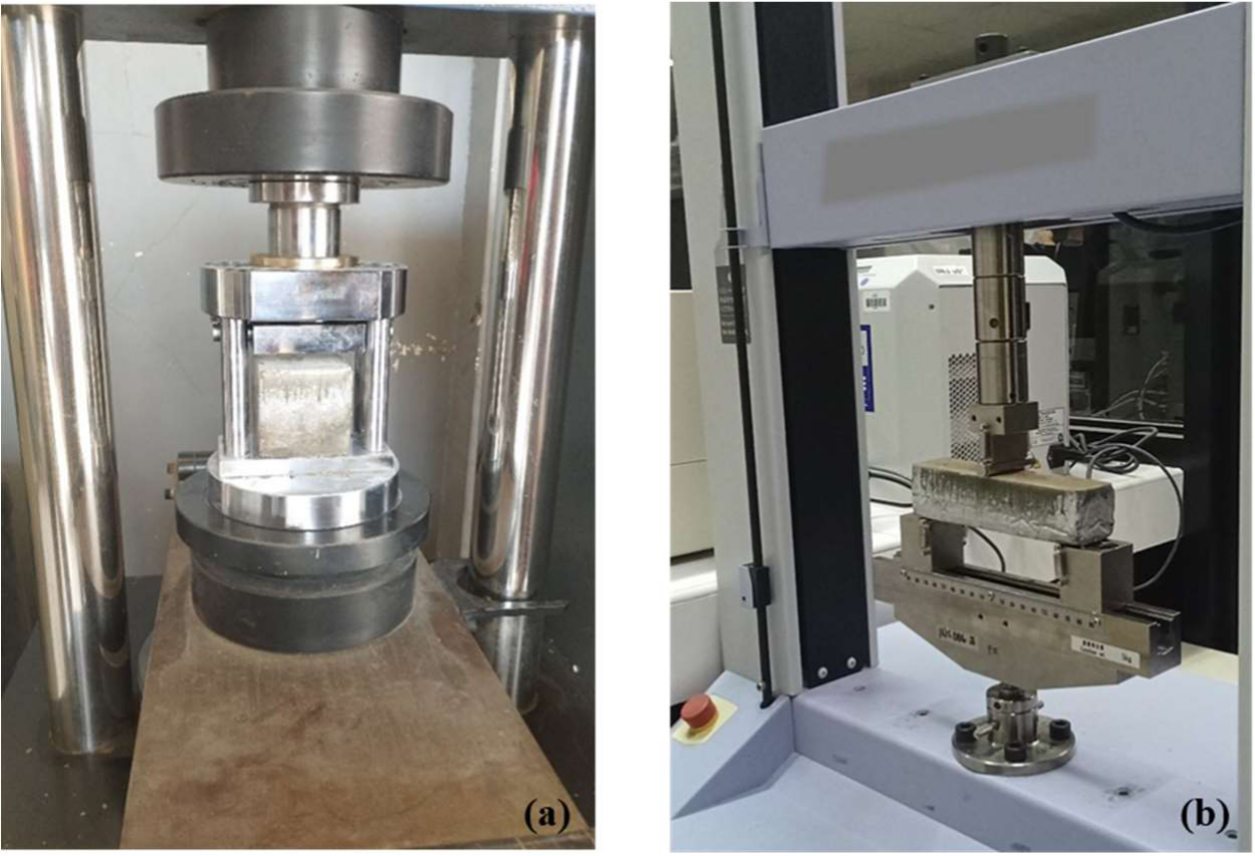
(a) Compression test of the concrete and (b) three-point bending test of the concrete.

Mechanical tests were conducted at the curing age of 28 days.^46^ Compressive strengths of cement mortar were measured according to ASTM C109,^47^ and the flexural strengths were determined using the three-point bending test following ASTM C348.^48^ Both tests were conducted using a universal testing machine from the Shimadzu AGS-X Series (Shimadzu Corp., Kyoto, Japan) (Figure 3).

#### 2.2.3 Fourier Transform Infrared Spectroscopy (FTIR)

To investigate the presence of the different functional groups in the PUMC, a Fourier transform infrared spectrometer (FTIR, Shimadzu ATR-FTIR IRTracer-100, Kyoto, JPN) was utilized in the 400–4000 nm wavelength range.

#### 2.2.4 Thermogravimetric Analysis (TGA)

The thermal decomposition profile was analyzed using a PerkinElmer TGA4000 thermogravimetric analysis (Waltham, MA) with a heating rate of 5 °C/min, sample size of 50 mg, nitrogen flow of 20 mL/min, and temperature range of 30–900 °C.

#### 2.2.5 Scanning Electron Microscopy (SEM) and Energy-Dispersive X-ray Spectroscopy (EDX) Tests

The surface morphology, microstructure, and elemental composition of the synthesized PUMC were assessed using a scanning electron microscope (SEM, JEOL JSM-6510LA, Tokyo, Japan) coupled with energy-dispersive X-ray spectroscopy (EDX). All samples were crushed and secured by using carbon tape. Since the PUMC is nonconductive, the samples underwent sputter-coating with gold for 1 min before the SEM and EDX analysis to ensure proper observation of the morphologies and microstructures.

#### 2.2.6 Abrasion Test

The abrasion test was conducted after 7 days of curing using an abrasion setup based on ASTM C944. The average material/weight loss was obtained after subjecting the PUMC samples to the abrasion test at 200 rpm and a normal load of 10 N for 2 min, with a minimum test schedule of three 2 min periods under the normal load.

## 3 Results and Discussion

### 3.1 PUMC Formation Mechanism

#### 3.1.1 Mechanism of Formation

Several studies have used polymers as mortar and concrete admixtures to alter the properties of the resulting cement mortar composite.^49−53^ Most of these studies have only probed the effects of the added polymer to the physicochemical^54−57^ and mechanical properties^31,58,^ of the resulting modified mortars and concrete. Although this is of fundamental interest, understanding the interplay between the cement and polymer phases at the molecular level will give more insight into how and why the admixture can impact the properties and performance of the resulting composite product. Thus, this aspect is highlighted in this paper. The mechanism of cement hydration is a well-established process consisting of a series of chemical reactions that take place with the addition of water.^60,61^ The hydration of the complex compounds in cement results in the formation of calcium silicate hydrate (C–S–H), the most relevant binding phase in the cement mortar matrix,^61^ calcium hydroxide (Ca(OH)_2_), calcium carbonate (CaCO_3_), and other hydration products like ettringite. On the other hand, the chemistry of PUs is entrenched in the science of polymers, resulting from the reaction between polyols and isocyanates.^62^ However, the combination of cement hydration and PU film formation to produce PUMC has not been thoroughly studied from a mechanistic standpoint. A general multistep model for liquid resins and prepolymers has been proposed by Ohama, describing the simultaneous hydration and polymerization reactions that produce an interpenetrating polymer network (IPN) within the mortar and cement mortar matrix.^63^ To supplement this model with more details, the specific interactions between the PU and cement phases of the composite were investigated.

**Figure 4:**
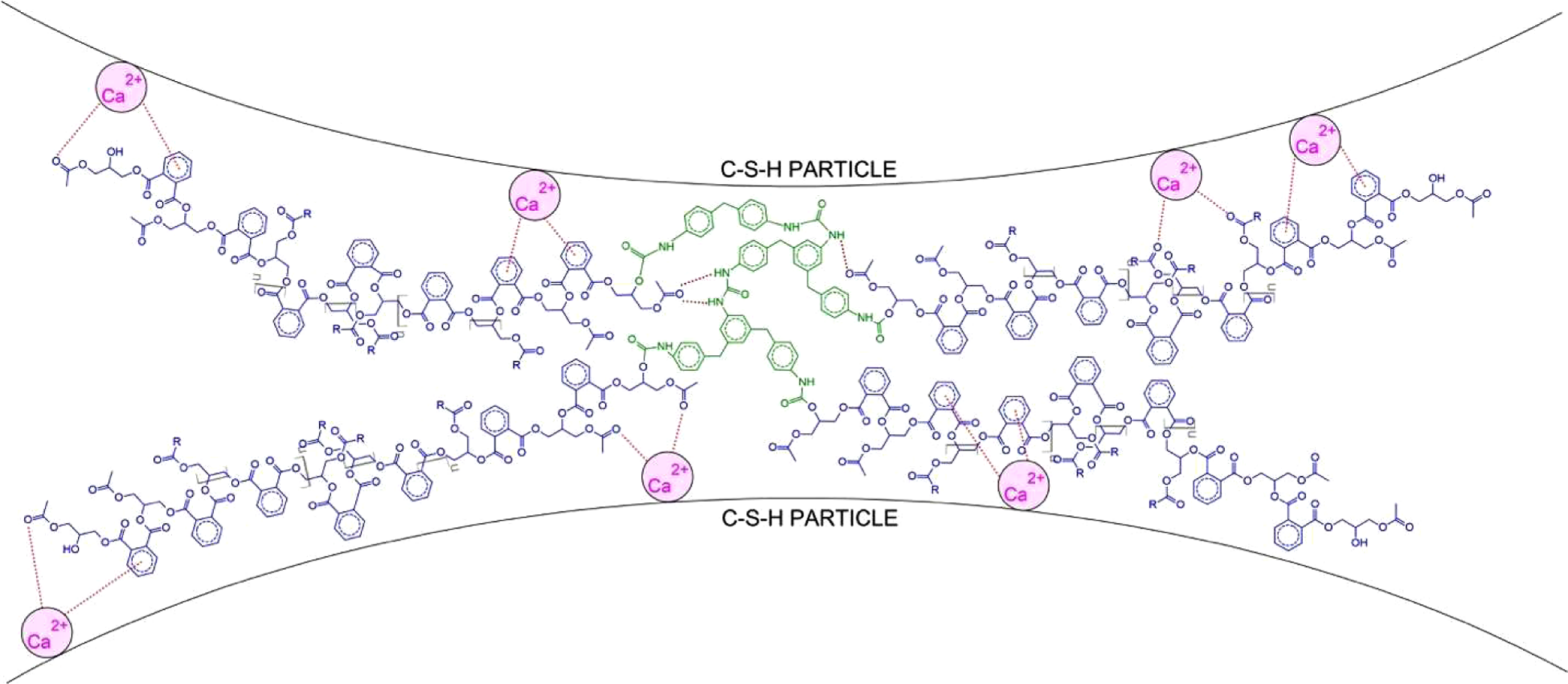
Proposed formation mechanism of the polyurethane-modified cement mortar (PUMC) highlights the coordination interaction of the mortar and polyurethane (PU) phases.

Figure 4 shows the interaction between the cement hydrates and the PU matrix. It is posited that the primary interaction governing the incorporation of PU into cement mortar is through the formation of coordination complexes. This is due to the abundance of calcium (Ca^2+^) ions during cement hydration that act as the central ion and interact with the amine-, carbonyl-, and phenyl-rich PU that acts as the complexing agent, also known as the ligand. This notion is supported by another study that elaborates on the facile coordination of Ca^2+^ ions and superplasticizers with complexing groups.^64^ Although considered a noncovalent interaction, the metal–ligand coordination between the composite phases has strong enough binding energies to reinforce the composite material and improve its mechanical properties.^65^ Moreover, a noteworthy characteristic of the PU admixture is the presence of multiple functional groups that readily coordinate with metal cations, constituting a polydentate ligand.^66^ Aside from acting as ligands, the aromatic groups in the PPP prepolymer also offer further mechanical advantage due to their excellent stability and strength compared with their aliphatic equivalents.^67^

#### 3.1.2 Chemical Structure Analysis

**Figure 5:**
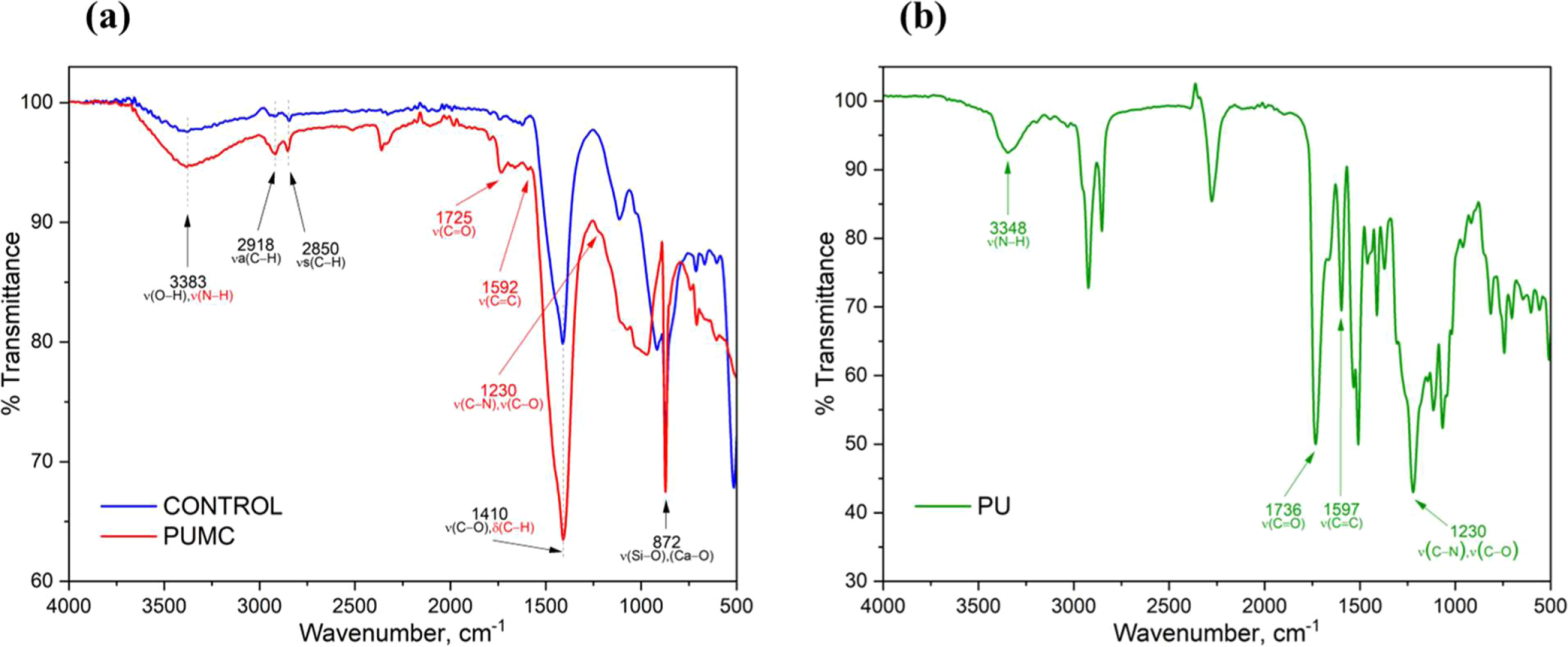
FTIR spectra of (a) the unmodified concrete (control) and the polyurethane-modified cement mortar (PUMC), and (b) the polyurethane (PU) admixture of PUMC.

The PU modification of cement mortar resulted in a change in the chemical composition of the cement mortar matrix, as evidenced by the samples’ FTIR spectra. Figure 5a compares the IR spectra of the unmodified cement mortar (control) and PUMC, while Figure 5b features the spectrum of PU. A few of the key transmission bands in the IR spectrum of cement mortar are shown in Figure 5a. The markings in black indicate the groups associated with cement hydration products, all of which are found in the spectrum of the control and PUMC. The O–H stretching band at PU 3383 cm^–1^ corresponds to the hydroxyl groups of C–S–H.^68^ The asymmetric and symmetric stretching vibrations of the C–H groups are observed at 2918 and 2850 cm^–1^, respectively. Furthermore, the band at 1410 cm^–1^ indicates the presence of C–O groups in carbonate in both the control and PUMC samples. Lastly, the sharp peak at 872 cm^–1^ is attributed to the stretching bands of Si–O and Ca–O of C–S–H in both samples.

Despite the concordance between the IR bands of the control and PUMC, some distinctions can be noticed in PUMC, not only in terms of peak intensity but also in the presence of new peaks. These differences can be correlated to the key transmission bands in the spectrum of PU in Figure 5b. The intensity of the O–H peak is greater in PUMC than in the control. This is due to the presence of N–H groups in PU at 3348 cm^–1^. The shift in the transmission band of N–H from 3348 cm^–1^ in PU to 3383 cm^–1^ in PUMC is an indication of the presence of intermolecular forces, specifically H-bonding between the urethane groups and nearby functional groups capable of H-bonding. This is indicated in Figure 4. The C–H band of PUMC at 2918 and 2850 cm^–1^ also showed a considerable increase in peak intensity compared with the control, owing to the abundance of C–H groups in the PU. The bending vibrations of these C–H groups at 1410 cm^–1^ also lead to greater peak intensity in the PUMC spectrum.

Moreover, three unambiguous peaks can be observed in the spectrum of PUMC but are absent in the spectrum of the control cement mortar sample. The first IR peak at 1725 cm^–1^ may be attributed to the stretching vibration of carbonyl groups in the PU admixture. The same band can be seen in Figure 5b for the IR spectrum of PU itself at 1736 cm^–1^, showing a shift toward lower wavenumbers when incorporated into cement mortar. The second peak at 1592 cm^–1^, denoting the stretching vibration of aromatic C=C (phenyl ring), also showed a downward shift compared with its corresponding peak in PU at 1597 cm^–1^. This notable shift in the absorbance peaks of the carbonyl and phenyl groups in the PUMC spectrum is indicative of its interaction within the cement mortar matrix.^69^ This interaction is inferred to be that of metal coordination between these groups and the Ca^2+^ cations that are predominant in the cement mortar mix, as represented in Figure 4. Lastly, a band in PUMC can be found at 1230 cm^–1^ attributed to the stretching vibrations of the C–N bond in urethane and the C–O bond in aromatic esters, which are absent in the control. The differences between the IR spectrum of PUMC and the control indicate successful consolidation of PU in cement mortar. At the same time, the shift in the PUMC peaks corresponding to the functional groups in PU supports the postulated intermolecular interaction between the polymer and cement mortar.

#### 3.1.3 Thermal Analysis

**Figure 6:**
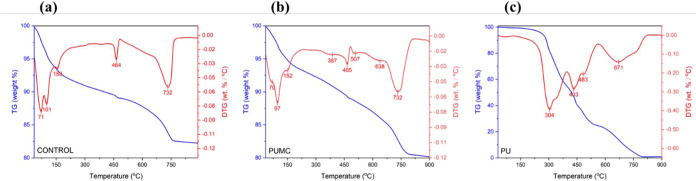
TGA and DTG curves of (a) unmodified concrete (control), (b) polyurethane-modified cement mortar (PUMC), and (c) PU admixture of PUMC.

The thermal performance of PUMC is studied to understand the influence of PU addition on the behavior of the cement-mold matrix under thermal stress. Figure 6 compares the TG and DTG thermograms of PUMC with those of the unmodified cement mortar and PU.

Figure 6a illustrates five distinct DTG peaks indicative of the degradation of different cement mortar components in the unmodified cement mortar control sample. The first DTG peak at 71 °C, the second at 100 °C, and the third at 158 °C represent the mass losses from the removal of water and the degradation of several hydration products such as C–S–H gel, ettringite, and C_2_ASH_8_.^70,71^ The fourth sharp peak at 464 °C indicates the decomposition of Ca(OH)_2_ produced after curing of the cement mortar.^72^ The most pronounced peak at around 732 °C is characteristic of the thermal decomposition of CaCO_3_.^73^ These degradation peaks of the unmodified cement mortar (Figure 6a) can also be discerned in the thermogram of PUMC in Figure 6b. The presence of these peaks signifies that the samples underwent proper curing.

Moreover, the thermogram of PUMC in Figure 6b noted a few new degradation peaks that did not resemble that of the unmodified cement mortar control sample. Specifically, three new peaks are apparent in Figure 6b at 387, 507, and 638 °C, which can be attributed to the PU admixture in the PUMC sample. When compared to the thermogram of PU in Figure 6c, a certain degree of congruence can be inferred. The first degradation peak of PU at 304 °C can be attributed to the breakdown of urethane linkages in the polymer matrix.^74^ The second and third peaks at 433 and 483 °C, respectively, fall within the range of soft segment decomposition, wherein the polyol component of PU degrades. Lastly, the peak at 671 °C shows the degradation of the isocyanate components of PU. These points roughly correspond to the three peaks in the PUMC thermogram. However, the PUMC degradation temperatures for the urethane linkages and polyol are noticeably higher than for PU. This substantial increase may be attributed to the interfacial interaction between the cement mortar matrix and the polymer that imparts some level of thermodynamic stability.^52^ The decrease in the degradation temperature of the isocyanates, on the other hand, may be owed to the heat barrier effect, wherein the cement mortar layers accelerate the degradation process due to accumulated heat.^75^ These changes in the thermal behavior of the PU phase within the cement mortar matrix support the interaction between the admixture and the cement mortar matrix.

### 3.2 Mechanical and Physicochemical Properties

#### 3.2.1 Compressive and Flexural Strength

The mechanical properties of PUMC are paramount to assessing its suitability for construction applications, such as in industrial flooring systems. Thus, the effects of any modification on the mechanical performance must be sufficiently investigated.

**Figure 7:**
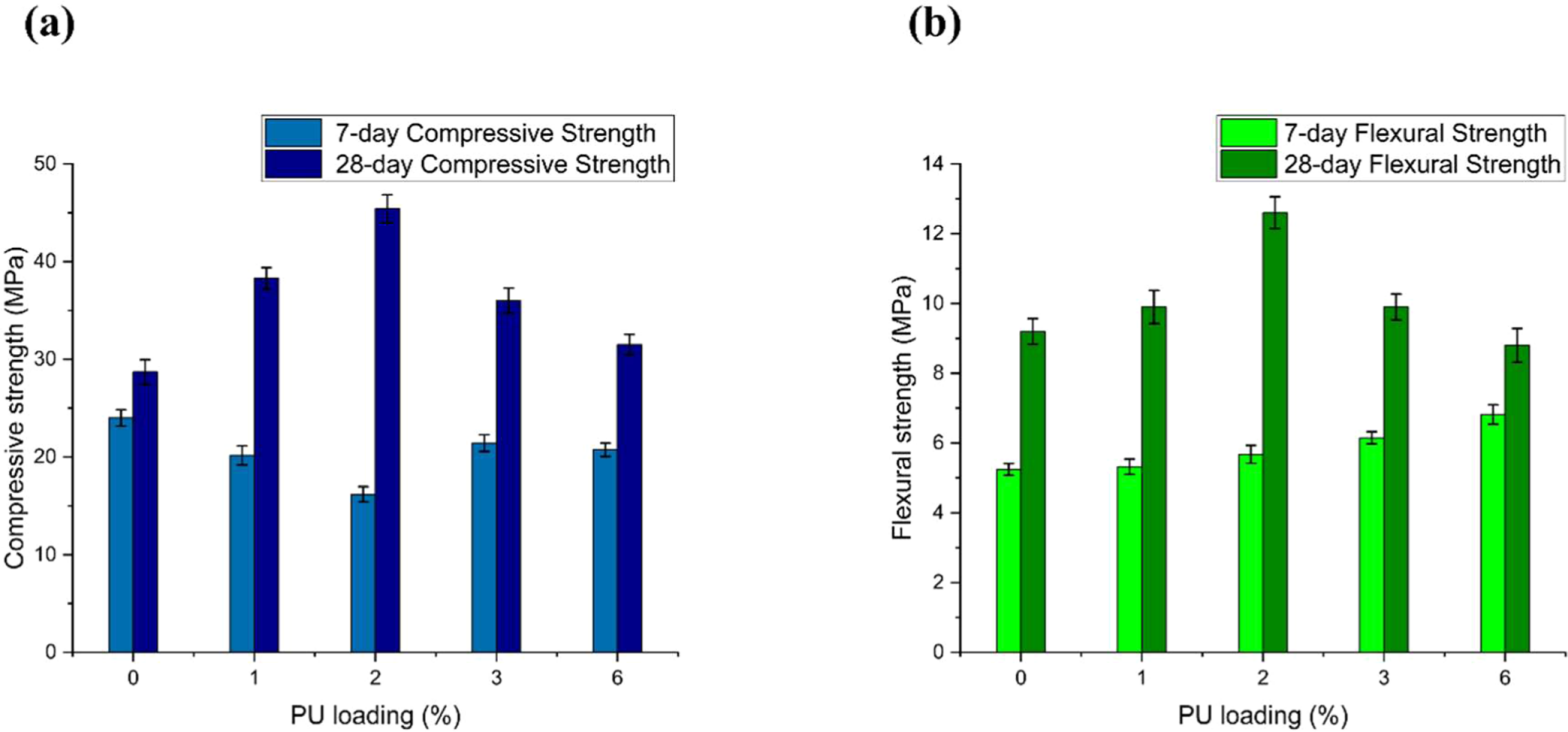
(a) Compressive and (b) flexural strengths of mortar samples at different polyurethane (PU) admixture loading after 7-day and 28-day dry curing periods.

The reduction of the 7-day compressive strength of the PUMC is consistent with the results of other PMC studies^31,39^ wherein the incorporation of the polymer admixture prolongs the curing time, thereby negatively impacting the early age strength of the material. On the other hand, the effects of PU addition on the 28-day strength of PUMC, as shown in Figure 7, highlight an interesting trend. The highest increase in both compressive and flexural strength was recorded at a 2% PU loading. This records a 58.2% increase in compressive strength and a 37.0% increase in flexural strength relative to the unmodified cement mortar control sample with 0% PU. When the PU loading is further increased to 3%, the compressive and flexural properties shift negatively. This decline was verified when the PU loading was doubled at 6%. Nevertheless, the lowest recorded compressive strength of PUMC at 6% loading is still 9.8% greater than the strength of the control, while the flexural resistance of PUMC showed a reduction slightly below that of the control sample with 0% PU.

The improvement in the mechanical properties of PUMC at lower PU loadings (<3%) is attributed to the stiffening effect imparted by the PU admixture, specifically its interaction with cement mortar. On the macroscopic level, the PU molecules adhere to the surface of the C–S–H matrix. Following the mechanistic description of Ohama et al. that the mechanical improvement is due to the IPN firmly holding the aggregates together,^42^ the layer of PU film on the surface of the cement mortar particles fills in the voids and cracks in the cement mortar, thus improving the pore structure and compactness of the resulting composite. On the microscopic level, the metal–ligand coordination forces between the cement mortar and PU shown in Figure 4 offer new insight into the molecular aspect of reinforcement that the polymer admixture provides. Additionally, the stability and strength imparted by the aromatic structure in the PU admixture are presumed to also contribute to these improvements. The combination of the macro- and microscale interplay between the cement mortar matrix and the PU admixture bolsters the mechanical properties of the PUMC. Thus, when the sample is subjected to external stresses, the polymer admixture helps increase its resistance before failure.^61−63^

Moreover, the decrease in the compressive and flexural strengths of PUMC at PU loadings greater than 2% may be due to the excess admixture failing to form a continuous composite structure with the cement phase. Without the formation of proper IPN, the PU admixture will tend to form clusters that act as microaggregates. These discrete polymer clusters, being significantly softer than the cement phase, behave similarly to voids or cavities that disrupt the PUMC structure’s continuity and make it more susceptible to the development and proliferation of microcracks.^76^ This will ultimately result in reduced mechanical properties.

#### 3.2.2 BET–BJH Analysis

**Figure 8:**
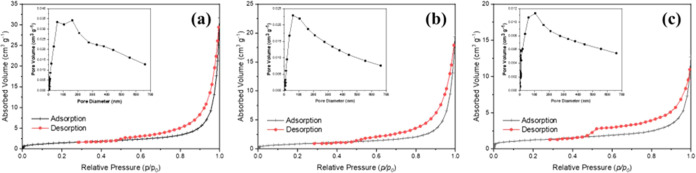
Nitrogen adsorption–desorption isotherms, and insets are the corresponding pore size distribution of (a) Control (0% PU), (b) 2% PU loading, and (c) 6% PU loading.

The specific surface area and pore structure of control sample (0% PU), 2% PU loading, and 6% PU loading were analyzed using N_2_ adsorption–desorption techniques. In Figure 8a–c, the N_2_ adsorption–desorption isotherms and corresponding pore size distributions of the synthesized curves of different samples are depicted. All samples exhibit similar sorption isotherms and pore size distributions. The BET surface area, pore volume, and mesopore volume of the various samples are summarized in Table 6.

**Table 6:** Surface Area, Total Pore Volume, and Mesopore Volume of Control (0% PU), 2% PU Loading, and 6% PU Loading

sample	BET surface area (m^2^/g)	total pore volume (cm^3^/g)	mesopore volume (cm^3^/g)
control	5.5059	0.033612	0.045502
2%	3.2913	0.022956	0.029685
6%	4.3107	0.014274	0.018063

BET surface area and pore volume results reveal significant information about the porosity and mechanical properties of PUMC. The control sample had the highest BET surface area, having a more porous microstructure in comparison with PU-loaded samples. Upon the introduction of 2% PU, a substantial decrease in BET surface area to 3.2913 m^2^/g was observed, suggesting that pore-filling and blocking were thus effectively caused by the PU additive. In contrast, the 6% PU sample exhibited an increased BET surface area of 4.3107 m^2^/g compared to the 2% PU sample but remained below the control, which may indicate additional features of the microstructure introduced by the higher content of PU.

The total pore volume data thus support these findings, wherein the control sample has the highest pore volume of 0.033612 m^3^/g, indicative of higher porosity. It follows that at both 2% PU and 6% PU, there is a drastically reduced pore volume of 0.022956 m^3^/g, already reaching nearly the density of the totally filled sample of 6% PU, which had the lowest level of pore volume at 0.014274 m^3^/g.

In cementitious materials, porosity strongly influences mechanical properties, such as compressive and tensile strengths. A denser microstructure typically correlates with a higher compressive strength, as anticipated for the 2% PU sample due to its reduced porosity indicated by BET surface area and pore volume data. Conversely, despite its lower pore volume, the 6% PU sample’s higher BET surface area suggests a nuanced relationship between pore structure and mechanical strength.

The 2% PU sample demonstrates optimal balance, showing the highest compressive strength, likely attributable to reduced porosity and enhanced structural integrity. In contrast, while the 6% PU sample exhibits lower pore volume, potential microstructural changes may slightly diminish its compressive strength compared to the 2% PU sample yet still surpass the control.

Regarding tensile strength, the 2% PU sample exhibits superior performance due to enhanced bonding within the mortar matrix, whereas the more porous control maintains higher flexibility compared to the denser 6% PU sample, resulting in higher tensile strength for the control.

#### 3.2.3 Self-Leveling Properties

**Figure 9:**
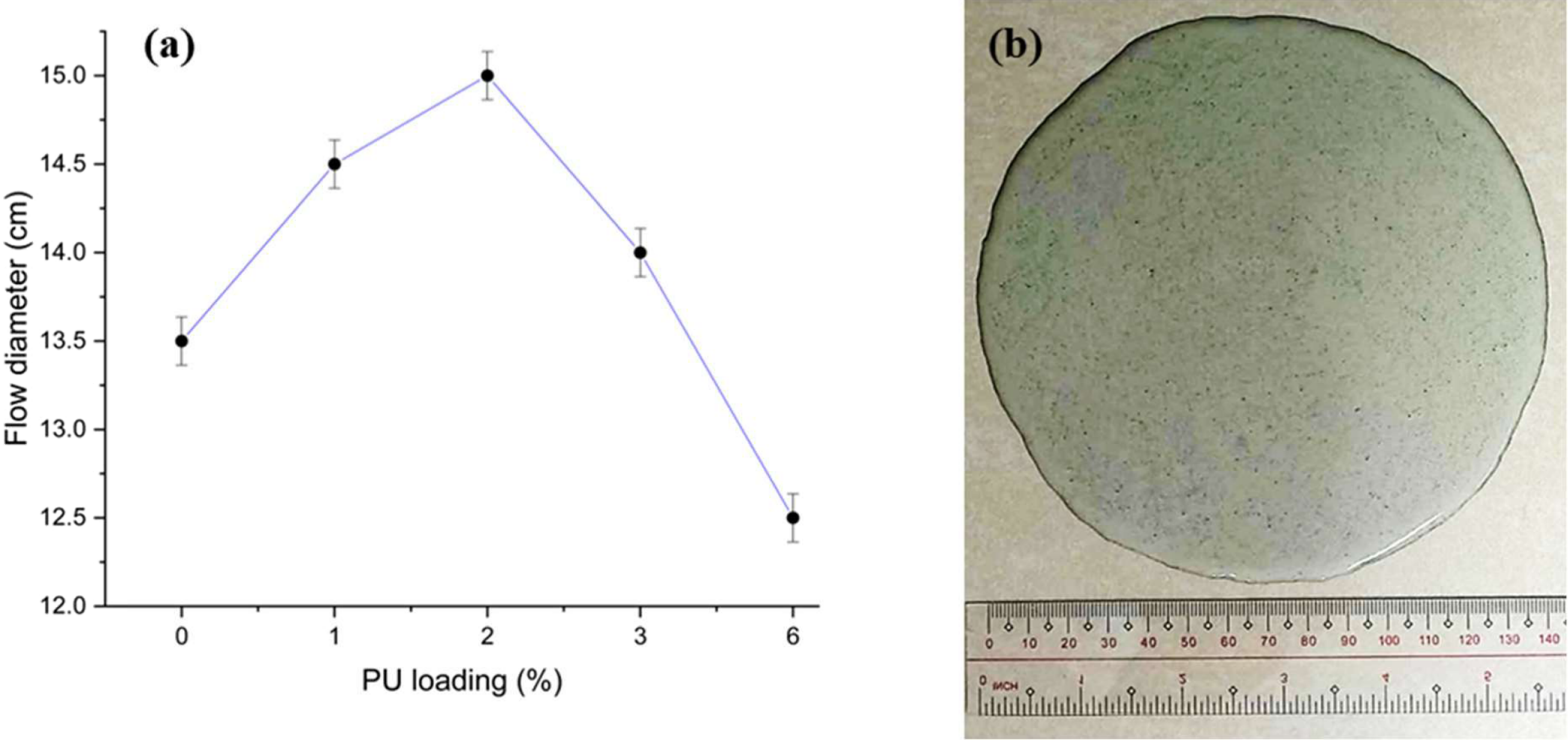
(a) Flow diameters of the concrete samples at different polyurethane (PU) admixture loading; (b) measurement of the flow diameter of a PU-modified concrete (PUMC) sample with 3% PU loading.

A distinct characteristic of PMC flooring materials is its self-leveling property, which refers to the properties of the flooring materials that spread out evenly and settle into a flat, level surface without needing external manipulation or assistance. This physicochemical property is imparted to the modified cement mortar by the SP, allowing for high flowability without the need for excessive amounts of water. Since the PUMC samples are engineered for this application, the self-leveling attribute of the samples is evaluated using the initial flow test in ASTM C1708.^77^ The flow test results of the control and PUMC samples with varied PU loading are reported in terms of the flow diameter in Figure 9.

The data in Figure 9a indicate an optimum PU admixture loading at 2% to improve the initial flow diameters of the PUMCs. This amount coincides with the optimum amount of the PU admixture to obtain the highest increase in the mechanical strengths of the modified cement mortar samples, specifically at 2% PU loading. This excellent influence of the PU admixture on the mechanical and physicochemical properties of the PUMC is a good indicator of its compatibility with the cement mortar components. It is also worth noting that the uniform improvements in the PUMC properties at the same PU loading are unlike most existing studies, wherein the best modified cement mortar properties are attained at different PU loading for each property.^78^ This will inevitably lead to a targeted approach in property enhancement but at the expense of another characteristic. However, based on the results of this work, the self-leveling, compressive, and flexural strength properties were all augmented, achieving optimal material improvement.

Further increase in the PU loading leads to a significant decline in the self-leveling property of the PUMC samples.^79^ This is due to the increase in viscosity of the PUMC mixture from the excess incorporation of the PU admixture.^80^ The increase in viscosity is connected to the increased likelihood of the MDI component of PU reacting with water in the mortar mix. This reaction produces hard urea segments that set the slurry, thus reducing flowability. Nevertheless, the flow test results in Figure 9 show that the PUMC samples still maintained their self-leveling characteristics despite the slight decrease since the diameters of the flow test samples exceeded the 12.5 cm threshold to be considered self-leveling, as per ASTM C1708.^81^

#### 3.2.4 Abrasion Resistance

**Figure 10:**
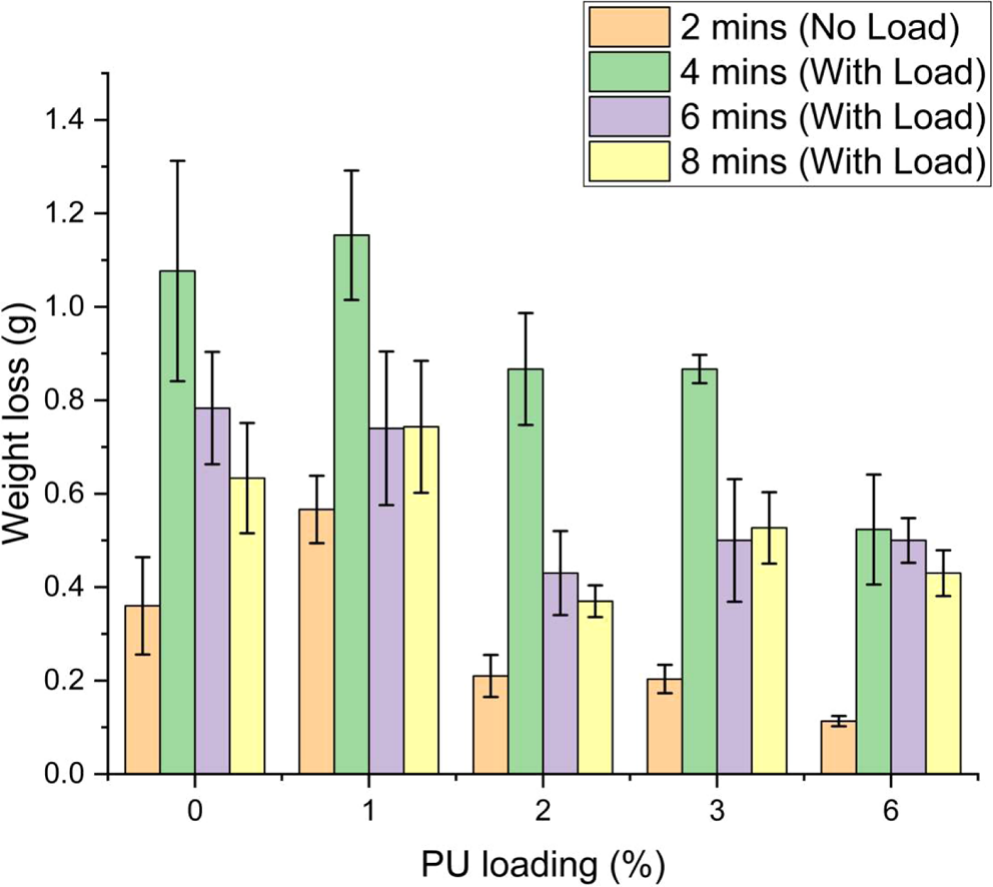
Weight loss of PUMC samples with varied PU loading during abrasion resistance testing.

Abrasion resistance is a critical property of industrial flooring materials, especially in environments subjected to heavy foot traffic, machinery, and frequent cleaning such as manufacturing plants, warehouses, commercial kitchens, and healthcare facilities. The results of the abrasion resistance test per ASTM C944^79^ conducted on the PUMC samples are presented in Table 7 and Figure 10.

**Table 7:** Abrasion Test Results for PUMC Samples

	abrasion loss at 7 days dry curing (g)			
specimen	2 min (no load)	4 min (with load)	6 min (with load)	8 min (with load)
0%PU/cement	0.3600	1.0767	0.7833	0.6333
1%PU/cement	0.5663	1.1533	0.7400	0.7433
2%PU/cement	0.2100	0.8667	0.4300	0.3700
3%PU/cement	0.2034	0.8667	0.5000	0.5267
6%PU/cement	0.1133	0.5233	0.5000	0.4300

The abrasion test results in Figure 10 show that the highest weight loss occurred upon application of the 10 N load during the abrasion test. This is due to the surface layer of the mortar being less resistant to abrasion as it is mostly composed of polymer and cement. As the test duration increases, more fine aggregate is exposed and the rate of abrasion loss is decreased.^82^ The sample containing 6% PU by weight of cement showed the lowest total abrasion loss, followed by the 2% PU sample. Both 6% PU and 2% PU PUMC samples exhibited higher abrasion resistance versus the control cement mortar sample, showing improvements of 18.2 and 6.20%, respectively.

### 3.3 Microstructure

**Figure 11:**
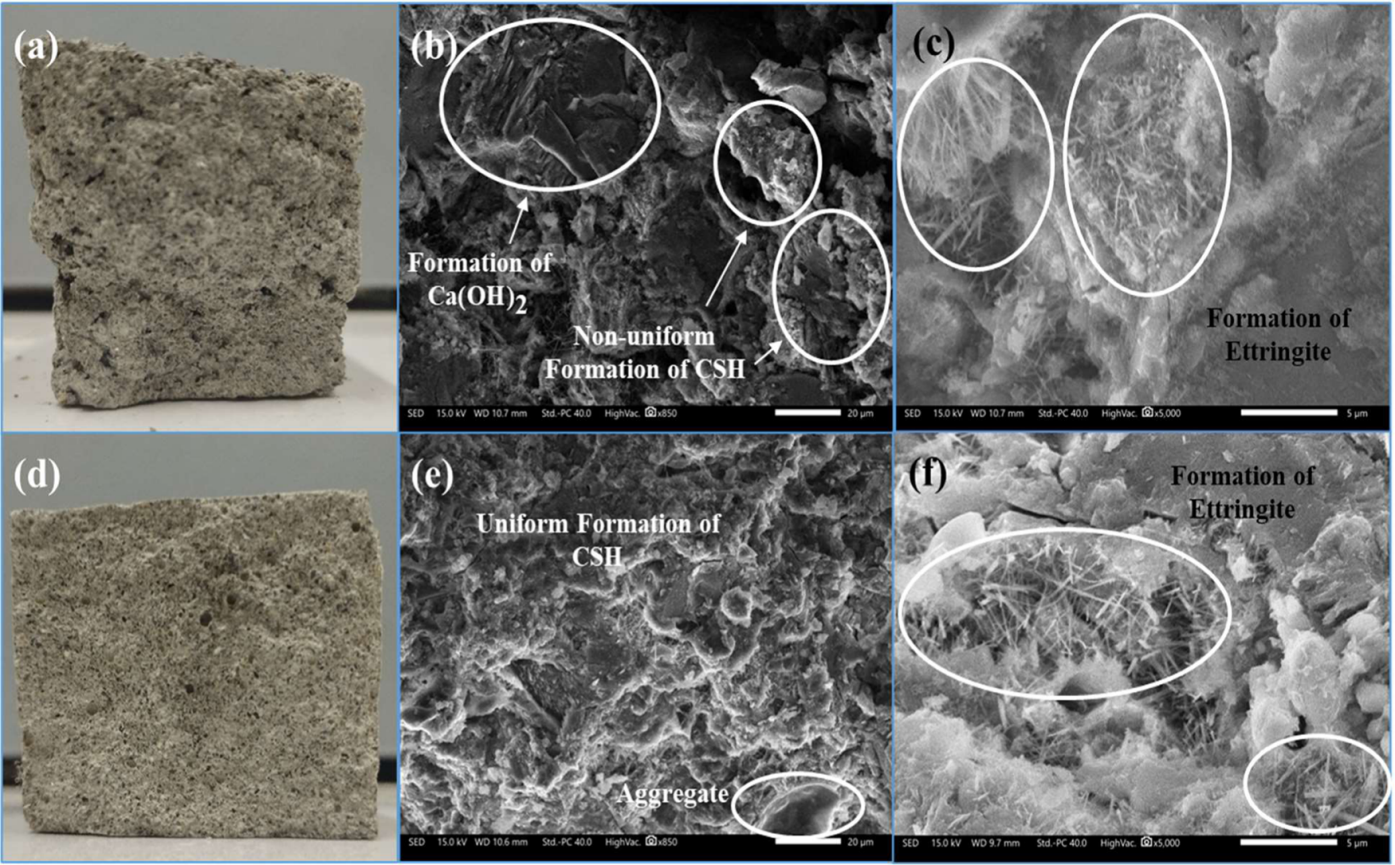
(a) Cross section of unmodified concrete control sample with 0% polyurethane (PU) at its fracture point after the bending test showing a nonuniform particle and pore distributions; (b, c) SEM micrographs of the control sample featuring the formation of cement hydration products; (d) cross section of polyurethane-modified concrete (PUMC) at its fracture point after the bending test showing a relatively uniform particle and pore and distributions; (e, f) SEM images of PUMC with 2% PU loading featuring the improved surface morphology and microstructure, and the formation of cement hydration products.

**Figure 12:**
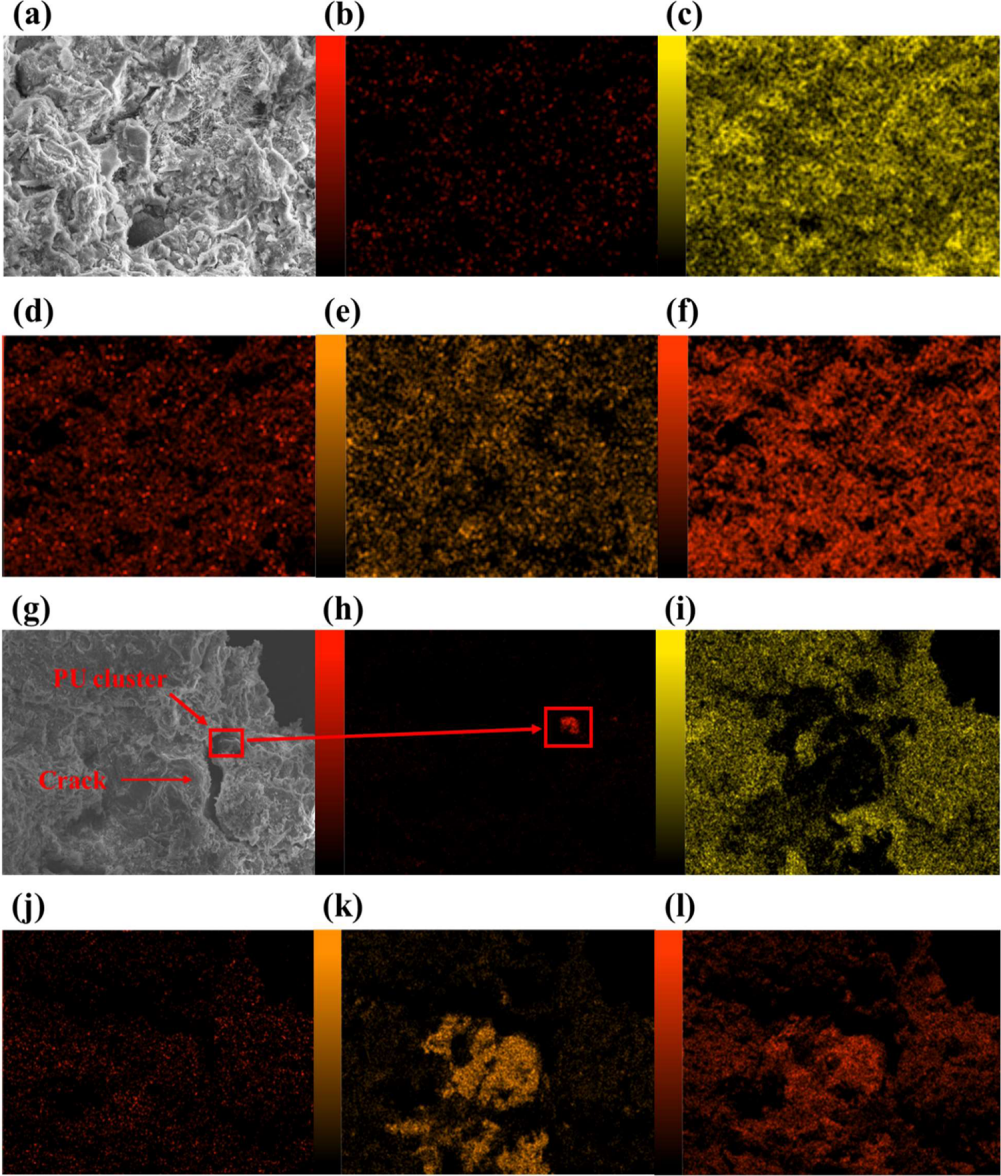
(a) SEM image of the polyurethane-modified concrete (PUMC) with 2% PU loading; (b) EDX map of the carbon, (c) calcium, (d) nitrogen, (e) silicon, and (f) oxygen contents in the 2% PUMC sample. (g) SEM image of PUMC with 6% PU loading; (h) EDX map of the carbon, (i) calcium, (j) nitrogen, (k) silicon, and (l) oxygen contents in the 6% PUMC sample.

Examining the microstructure of cement mortar is paramount for the proper assessment of its composition and homogeneity as well as the detection of defects and structural anomalies. These factors ultimately determine the hardened properties, specifically, the mechanical strength of the PUMC samples. The results of the SEM–EDX imaging for the fabricated samples are presented in Figures 11 and 12.

Figure 11a–c shows the uneven surface morphology of the control sample’s cross section, while Figure 11d–f reveals a relatively homogeneous morphology of the PUMC at both fracture points after compression. It can be observed that the structure of the control in Figure 11a displays a degree of particle segregation, which is a common problem in a regular cement mortar. This phenomenon is caused by the loosely packed hydration products depicted in Figure 11b. This loose packing affects the coherence between the hydration products during the curing process, ultimately leading to lower mechanical strength.^83^ Moreover, the formation of ettringite (needle-like structures) is shown closely in Figure 11c, indicating the proper setting of cement mortar during the dry curing process.^84^ In contrast, the PUMC sample in Figure 11d has a more even surface morphology in terms of particle packing and pore size distribution compared to the control. This is reflected in its micrograph in Figure 11e, which shows a surface morphology with considerable improvement in homogeneity and cohesion of hydration products compared to the unmodified control sample. Closer inspection of the PUMC sample also revealed the presence of ettringite, as shown in Figure 11f.

The images in Figure 11 illustrate physical evidence of the effects of PU modification through microstructural analysis of the PUMC samples. Congruently, the observed differences in the morphology between the control and MC samples support the improvements discussed previously in their mechanical and physicochemical properties. From a chemical standpoint, the favorable influence of the PU admixture on cement mortar predicates the proposed PUMC formation mechanism, wherein the cement mortar phase and the PU phase of the comatrix were reinforced by the metal–ligand coordination between Ca^2+^ ions in cement mortar and complexing agents in the PU. Moreover, it can be inferred that the microstructural and elemental characteristics of the PU admixture have positively affected the properties of PUMC.^39^

Furthermore, since the IPN in the composite material cannot be distinguished from the SEM micrographs alone, it is supplemented with EDX microanalysis in Figure 11 to examine the distribution of elements in the sample. Figure 11a–f shows the micrograph and EDX maps for various elements of PUMC with 2% PU loading, while Figure 11g–l shows the corresponding images for the PUMC sample with 6% PU loading. Aside from the typical elements constituting cement mortar, such as calcium, silicon, and oxygen depicted in Figure 11c,e,f, respectively, carbon and nitrogen were also identified in the analysis shown in Figure 11b,d, respectively. The even distribution of these elements in the PUMC sample with 2% PU loading is indicative of the successful consolidation of the PU admixture and the formation of an evenly dispersed IPN in the composite. Such observations align with the required microstructural uniformity to positively influence the modified cement mortar’s mechanical and physicochemical properties.^85^

Contrarily, the EDX maps pertaining to the PUMC sample with 6% PU loading in Figure 12g–l offer a clear depiction of the effects of excess PU loading and give morphological insight into the observed negative trend on the properties of PUMC when the PU loading is further increased. The most noteworthy detail is the presence of a crack in the microstructure of 6% PUMC in Figure 12g. Directly at the edge of this crack is a clump of carbon detected in Figure 12h, indicating the agglomeration of the PU admixture and its failure to form an IPN. This unsuccessful IPN formation may be ascribed to the increased likelihood of the MDI in PU reacting with water rather than with the PPP prepolymer. This is supported by the presence of dispersed nitrogen shown in Figure 12j. Additionally, the interference of MDI with the hydration process is also seen to have caused a certain degree of particle segregation, as revealed in Figure 12i,k,l, showing a relatively uneven distribution of calcium, silicon, and oxygen in the modified cement mortar, respectively. These findings collectively resulted in the performance deterioration of PUMC.

## 4 Conclusions

This study delved into the investigation of the chemistry and evaluation of the properties of a newly developed PUMC flooring material. A striking overall improvement of the material’s 28-day compressive and flexural strengths was recorded to attain a 58.2 and 37.0% increase, respectively. Similarly, the self-leveling capacity of PUMC also gained a 20.0% enhancement, in terms of its initial flow performance. These optimal results were all achieved with the addition of a 2% PU mixture. While the 6%PU specimen yielded the best results in the abrasion test. These enhancements were substantiated with morphological evidence that revealed the microstructural changes in PUMC associated with its performance, such as a relatively even particle and pore size distribution and uniform IPN formation analyzed by using BET–BJH and SEM–EDX analyses. In addition to the uniform IPN, the proposed metal–ligand coordination between Ca^2+^ ions in cement mortar and the ligand groups in PU strengthens the interfacial connectivity of the two phases via noncovalent binding. This is supported by FTIR and TGA analyses, which not only highlight the successful consolidation of PU and cement mortar but also indicate the presence of attractive forces between the two phases. Moreover, the deterioration of PUMC’s performance beyond the optimal PU loading was owed to the destructive effects of PU agglomeration and its interference with the cement hydration process. These findings demonstrate optimal material development through the overall improvement of PUMC’s properties relevant to its industrial flooring application and propound a potentially revolutionary bio-based PU admixture for the construction industry. The study was unable to examine the early age (3 days) mechanical strength, the setting time, the workability, the effect of other PU percent loadings, and the effect of different curing regimes on the development of the composite material. Further research on the thermal properties, water absorption, and chemical resistance of fabricated PUMC specimens is in progress.
